# Bloodstream infection outbreak caused by burkholderia cepacia complex: the role of genetic sequencing in the investigation

**DOI:** 10.1186/2047-2994-4-S1-P228

**Published:** 2015-06-16

**Authors:** C Schmitt, ALP Maciel, MM Baraldi, MMMB Simonetti, M Cantarelli, G Turcato, LFV Oliveira, Í Boszczowski

**Affiliations:** 1 Infection Control, Oswaldo Cruz German Hospital, Sao Paulo, Brazil; 2 Neoprospecta, Florianopolis, Brazil

## Introduction

*Burkholderia cepacia* complex is a group of at least 15 species of non-fermentative Gram negative bacteria, common in the environment. These agents are often associated with infections in Cystic fibrosis and immunosuppressed patients, as well with healthcare associated infection outbreaks.

## Objectives

We aimed to describe an outbreak investigation of bloodstream infection caused by *Burkholderia cepacia* complex (BSIBcc).

## Methods

A case control study was carried out to investigate a (BSIBcc) outbreak in a 357 beds private hospital in the city of Sao Paulo. Cases were defined as bloodstream infection caused by B. cepacia complex occurred among patients after 48 hours of admission. Controls were selected among patients that shared the same ward and staid at least seven days within the same month of the respective case. Case-control relation was 1:2. Visits to ICU and operatory block were performed. Environmental samples were obtained for culture and genetic sequencing.

## Results

Between 03/2012 and 07/2014 we identified 12 bloodstream infections occurred in different wards. Central venous catheter (OR 1,36; IC 1,15-1,67) and intavenous cisatracurium (OR 10,75 IC 1,67-68,89), were associated to BSIBcc. Visits to operatory block revealed problems related with cold chain used for cisatracurium storage and other thermolabile drugs (no established hygiene routine for freezer, psychotropic box and anesthesia trolley were evidenced). Reusable ice packages were not easily cleaned. Samples obtained from surfaces of cold chain for classical microbiology, did not yield positive. *Burkholderia cepacia* complex was identified by genetic sequencing in cooler, pharmacy refrigerator, and reusable ice packages. Control measures: hygiene routine implementing, acquisition of easily washable coolers and reusable ice single use. The last case was identified in 12/2013.

## Conclusion

*Burkholderia cepacia* complex is an important pathogen associated with nosocomial outbreaks. Environmental cultures using classical microbiology are not sensitive for identification of these organisms. Genetic sequencing presented as a promising tool for environment Bcc investigation in the healthcare setting.

## Disclosure of interest

None declared.

